# Early Diagnosis of Neuropathy in Leprosy—Comparing Diagnostic Tests in a Large Prospective Study (the INFIR Cohort Study)

**DOI:** 10.1371/journal.pntd.0000212

**Published:** 2008-04-02

**Authors:** Wim H. van Brakel, Peter G. Nicholls, Einar P. Wilder-Smith, Loretta Das, Pramila Barkataki, Diana N. J. Lockwood, on behalf of the INFIR Study Group

**Affiliations:** 1 Royal Tropical Institute (KIT), Amsterdam, The Netherlands; 2 School of Nursing and Midwifery, University of Southampton, Southampton, United Kingdom; 3 Division of Neurology, National University Hospital, Singapore; 4 Naini Community Hospital, Allahabad District, Uttar Pradesh, India; 5 TLM Hospital Faizabad, Faizabad District, Uttar Pradesh, India; 6 London School of Hygiene and Tropical Medicine, London, United Kingdom; Sabin Vaccine Institute, United States of America

## Abstract

**Background:**

Leprosy is the most frequent treatable neuromuscular disease. Yet, every year, thousands of patients develop permanent peripheral nerve damage as a result of leprosy. Since early detection and treatment of neuropathy in leprosy has strong preventive potential, we conducted a cohort study to determine which test detects this neuropathy earliest.

**Methods and Findings:**

One hundred and eighty-eight multibacillary (MB) leprosy patients were selected from a cohort of 303 and followed for 2 years after diagnosis. Nerve function was evaluated at each visit using nerve conduction (NC), quantitative thermal sensory testing and vibrometry, dynamometry, monofilament testing (MFT), and voluntary muscle testing (VMT). Study outcomes were sensory and motor impairment detected by MFT or VMT. Seventy-four of 188 patients (39%) had a reaction, neuritis, or new nerve function impairment (NFI) event during a 2-year follow-up. Sub-clinical neuropathy was extensive (20%–50%), even in patients who did not develop an outcome event. Sensory nerve action potential (SNAP) amplitudes, compound motor action potential (CMAP) velocities, and warm detection thresholds (WDT) were most frequently affected, with SNAP impairment frequencies ranging from 30% (median) to 69% (sural). Velocity was impaired in up to 43% of motor nerves. WDTs were more frequently affected than cold detection thresholds (29% versus 13%, ulnar nerve). Impairment of SNC and warm perception often preceded deterioration in MF or VMT scores by 12 weeks or more.

**Conclusions:**

A large proportion of leprosy patients have subclinical neuropathy that was not evident when only MFT and VMT were used. SNC was the most frequently and earliest affected test, closely followed by WDT. They are promising tests for improving early detection of neuropathy, as they often became abnormal 12 weeks or more before an abnormal monofilament test. Changes in MFT and VMT score mirrored changes in neurophysiology, confirming their validity as screening tests.

## Author Summary

Leprosy is the most frequent treatable disease of the peripheral nerves. Yet, every year, thousands of patients develop nerve damage as a result of leprosy. If this is detected and treated early, the prognosis is good. We conducted the largest prospective study on this topic to date to determine which test would detect nerve damage earliest. One hundred and eighty-eight leprosy patients were selected from centres in North India and followed for 2 years after diagnosis. Nerve function was tested at each visit using a battery of nerve function tests. A large proportion of the patients had or developed subclinical nerve damage that was not detected with the standard clinical tests (monofilaments and voluntary muscle testing). Sensory nerve conduction was the most frequently and earliest affected test, closely followed by the warm temperature perception test. They are promising tests for improving early detection of nerve damage, as they often became abnormal 12 weeks or more before the monofilament test became abnormal. Changes measured with the monofilament and voluntary muscle tests mirrored changes in more advanced electronic measures, confirming their validity as screening tests. These findings open the door to future improvement of the prognosis of nerve damage in leprosy.

## Introduction

‘Early detection improves prognosis’ is a general axiom in medicine, and in leprosy delay in detection is strongly associated with an increased risk of neural impairment at diagnosis [1]–[4]. In addition, nerve function impairment (NFI) already present at diagnosis has been found to be a strong predictor of the risk of further immunological reactions or episodes of sensory or motor neuropathy [5]–[7]. Around 10% of the 300,000 new leprosy cases registered every year have signs of sensory, motor or autonomic neuropathy at diagnosis. The highest rates of impairment were reported from Ethiopia (55%) [7], while studies in Thailand and Bangladesh reported rates of 18% and 12%, respectively [4],[8]. New neuropathy may develop both during and after effective multi-drug therapy [9]. A substantial proportion of people with leprosy-related nerve damage will have life-long functional and/or social disability [10]. Early detection and treatment of NFI is therefore seen as a top priority [11].

Assessment of sensory function of nerves affected by leprosy is typically done with the monofilament test (MFT). This test uses standardised, graded nylon monofilaments to monitor touch sensation on the hand palms and foot soles semi-quantitatively [12]. Motor function is monitored using the voluntary muscle test (VMT) [13],[14]. Both tests have been shown to be valid and reliable under various conditions [15]–[17].

Nerve conduction (NC) testing has been done in leprosy patients, but most studies were small and cross-sectional and involved mixed groups of new and treated subjects [18]–[26]. Ramakrishnan & Srinivasan tried to determine which electrophysiological test would best discriminate between normal and abnormal (median) nerve function in leprosy [24]. They found that the amplitudes of the distal sensory nerve action potentials (SNAP) were more reliable indicators of leprosy neuropathy than sensory nerve conduction (SNC) velocities.

The only studies comparing different tests of nerve function were those by Naafs & Dagne, Touw-Langendijk et al. and Samant et al. [26]–[28]. They did not find advantage in motor nerve conduction (MNC) over VMT or sensory testing with monofilaments or a combination of the latter with nerve palpation. Investigators in cross-sectional studies have concluded that NC studies were very useful and would potentially detect pre-clinical neuropathy [19],[21],[24],[26].

Quantitative sensory testing (QST) has opened up new possibilities for the study of sensory neuropathy [29],[30]. The most commonly used methods are thermal threshold testing and testing of vibration perception thresholds. Thermal testing assesses small, unmyelinated C-fibres that mediate warm sensation and small, unmyelinated and myelinated Aδ fibres mediating cold sensation [31]. Like nerve conduction, vibrometry assesses large, myelinated Aβ fibres [32]. QST has been used only occasionally and in cross-sectional studies [33],[34]. Vibrometry did not appear to have much additional advantage over more established methods of sensory testing. Abbot et al. and Wilder-Smith et al. used laser Doppler flowmetry for quantitative assessment of autonomic nerve function [35]–[37]. This method appeared very sensitive and detected widespread autonomic neuropathy in hands and feet of leprosy patients [38].

The INFIR cohort is a group of newly diagnosed patients with multibacillary leprosy (MB). They had monthly tests of nerve function using MFT, VMT, nerve conduction studies, thermal sensation and vibrometry. This cohort has enabled us to test the hypothesis that monofilaments and VMT are relatively insensitive methods for detecting nerve damage. Having a cohort of patients has also enabled us to test the hypothesis that nerve damage occurs over a long period and can be detected earlier if more sensitive methods are employed.

## Methods

Details of the methods have been published before [39]; only a brief summary will be given here.

### Design

A cohort study with 4-weekly follow-up for one year and 8-weekly follow-up during the second year.

### Study subjects

303 patients with MB leprosy, newly diagnosed at two referral hospitals in Uttar Pradesh, India, were included in the cohort. Patients who had a reaction or sensory or motor impairment at diagnosis were excluded from this analysis. The study subjects were at different stages of their disease, as reported earlier [39]. A brief summary is given in the Results section below.

### Outcome events

Outcome events for this analysis were sensory impairment (SI) and motor impairment (MI), as detected by the MFT and VMT (for definitions see Text S1). The techniques have been described elsewhere [39]. ‘New impairment’ of a neurological parameter was defined as ‘impairment which was not present at any earlier follow-up visit’. Thresholds for impairment calculations are in the Analysis section.

### Outcome measures

#### Nerve function test results and outcome events

1. Median values of test parameters
2. Percentage of patients testing positive (impaired) for a given measure or marker.

#### Early detection of sensory or motor impairment

3. Sensitivity and positive and negative predictive value of each test in predicting clinically significant NFI diagnosed with MFT or VMT

### Examination and treatment

A standardised history using a checklist was taken from all patients. All patients had a physical examination and a basic neurological examination (including reflexes, joint position sense and nerve palpation) on admission and repeated at each visit. Evidence (signs and symptoms) of Type 1 Reaction (T1R), Erythema Nodosum Leprosum (ENL) and peripheral neuropathy was carefully sought.

### Nerve function assessment (NFA)

The following techniques were used for measuring nerve function at each follow-up visit.

#### Motor nerve function

1. **Voluntary muscle testing** (VMT) using the 0–5 modified MRC scale [39].
2. **Grip dynamometry**, key pinch and pulp-to-pulp pinch testing
A dynamometer was made of a sphygmomanometer cuff inserted in a cylindrical cotton cover and inflated to a baseline pressure of 20 mmHg. Pinch strength was measured in a similar way using a neonatal sphygmomanometer cuff [40].
3. **Motor nerve conduction measurements** (MNC)
Nerve conduction parameters were measured using Neurocare 2000® EMG machines (BioTech Ltd., Mumbai). The testing room was maintained at around 26°C (confirmed using ambient thermometers). Patients were allowed to acclimatise for 15 minutes before testing. Monopolar surface recording electrodes and bipolar hand held stimulating electrodes were used to obtain the compound muscle action potentials (CMAP). All motor tests used the belly-tendon method. The calculated values for latency, amplitude and conduction velocity were stored in an Access database. Skin temperatures were measured electronically at the palmar wrist and dorsum of the foot before the onset of nerve conduction testing and the measurements corrected at the time of analysis using standard formulae [41]. The filter setting for motor nerve conduction was 3 Hz for low frequency and 10 kHz for high frequency. The sensitivity and sweep was set at 5 mV and 50 ms respectively. The abductor digiti minimi, abductor pollicis brevis and extensor digitorum brevis muscles were used to test the ulnar, median and peroneal motor nerves respectively. Stimulation was performed at two sites. The distal stimulation was 6 cm proximal to the active recording electrode for all nerves. The proximal stimulation site was 10 cm above medial epicondyle for ulnar, antecubital fossa for median and behind the fibular head for the peroneal motor nerve. The velocity was calculated between the distal and proximal stimulation sites using the onset of the evoked CMAPs, amplitudes were measured from negative to positive peak.

#### Sensory nerve function

4. Sensory testing was done with a standard set of Semmes-Weinstein monofilaments (MF) [42]
The monofilaments used were 200 mg, 2 g, 4 g, 10 g and 300 g. Normal thresholds were 200 mg for the hand and 2 g for the foot (excluding the heel) [43]. The test sites and scoring methods have been described elsewhere [39].
5. **Sensory nerve conduction measurements** (SNC)
SNAP parameters were measured bilaterally on 4 nerves (radial cutaneous, ulnar, median and sural) using the same equipment and procedures as described under MNC. Monopolar surface recording electrodes and bipolar hand held stimulating electrodes were used to obtain the sensory nerve action potentials (SNAP). All the sensory nerve conduction testing were antidromic. The filter setting was 20 Hz for low frequency and 2 kHz for high frequency. The sensitivity and sweep was set at 10 µV and 20 ms respectively. Responses were averaged up to 6 times, if needed. Ulnar and median SNAPs were registered over digit 5 and digit 2, respectively. The radial sensory potentials were registered at the base of thumb and the sural behind the lateral malleolus. Electrical stimulation was performed over the nerve 12 cm proximal from the recording site for all tests. The onset of the evoked SNAP was used to calculate velocities; amplitudes were measured from the negative to positive peak.
6. **Vibration Perception Threshold** (VPT) testing
VPTs were testing with a Vibrameter II®, Somedic, Sweden. Application force-controlled measurements of the VPTs in microns of skin displacement, using the method of limits (slowly increasing vibration amplitude, until the person tested indicates that (s)he can feel the vibration) were made. The test sites were the thenar and hypothenar eminences (soft tissue), for testing the median and ulnar nerve, respectively, the dorsal first webspace for the radial cutaneous nerve, the plantar surface of the big toe (posterior tibial) and the mid-lateral border of the foot (sural). All tests were done bilaterally.
7. **Thermal threshold testing**
Thermal thresholds were evaluated using a Thermal Sensory Analyzer (TSA II®, MEDOC, Israel). Warm and cold detection thresholds (WDT/CDT) were measured relative to a baseline thermode temperature of 32°C, using an algorithm called the ‘method of levels’ [44]. 10°C and 50°C were set as measurable limits of cold and warm perception, respectively. Test sites were the same as for vibrometry, described above.

### Analysis

The thresholds for impairment were determined from normative studies done as part of this project. From these, age, sex and centre-specific normal thresholds were calculated as the 97.5^th^ centile of the log-transformed data. Each measured value in individual patients was compared with the appropriate age, sex and centre-specific normal threshold (back-transformed to real values). No significant differences were found between left and right extremities, so assessments were pooled for left and right.

Subjects who developed a new outcome event were matched for sex, age group, leprosy type and length of available follow-up with a control who had not developed an outcome event prior to or in the six months following the ‘outcome event visit’ of the case. E.g., if a case had an outcome event at visit 4, then only subjects who were free of outcome events until visit 10 were eligible as matched control. However, most analyses were not matched on a one-to-one basis, but a sub-group of the cases is compared as a group with their matched control group. With the matching criteria used, it proved impossible to exclude controls with prior, ‘old’ NFI in one or more nerves. This resulted in relatively high levels of neural impairment even in the control group. Therefore, to examine trends over time, another control group was selected. This group of 16 subjects was free of clinically detectable NFI (by MFT and VMT standards) throughout the follow-up period. Data in the trend graphs examining onset of NFI are compared with these ‘NFI-free controls’, as well as with the relevant normal thresholds.

Analysis regarding onset of NFI was done on nerves without evidence of any old impairment (measured by MFT or VMT). Nerves of patients receiving steroids for skin reactions were excluded, even if they developed new NFI, as were nerves biopsied because of an outcome event earlier during the follow-up. For NC parameters, non-conducting nerves were included in the predictive value analyses, because they are often also detected as impaired on other tests.

Prevalence estimates are given as percentages. The significance of associations between categorical variables was tested using the Chi-squared or Fisher’s exact test. Differences between proportions were tested with the z-test for differences between proportions. The term ‘concordance’ is used to describe the direct agreement between the results of two tests in terms of ‘impaired’ and ‘not impaired’. Analyses were performed using Stata software, v.9.

### Ethical considerations

The study was approved by the ethics committee of the Central JALMA Institute for Leprosy, a major leprosy research centre of the Indian Council for Medical Research. No financial incentives were given to participants. Informed written consent was obtained from individual study subjects before inclusion in the study, using a standard consent form. Further details are available elsewhere [39].

## Results

Three hundred and three subjects were enrolled in the study. Their mean age was 32.8 years (range 12–60). Over 50% had grade 1 or 2 impairment and 36% were smear-positive. Twenty-one percent had an average BI of 3 or more and 9.6% had grade 2 (visible) impairment of eyes, hands or feet. Thirty percent reported a detection delay of <6 months; 32% between 7–12 months and the remainder (38%) 13 months or longer. Of the 303 subjects, 115 had a reaction or NFI event at registration, leaving a cohort of 188 for the prospective ‘early detection’ analysis. Of these, 74 developed an outcome event (reaction or NFI) during the two-year follow-up (39%). For 73 of these cases, a matched control was found. The characteristics of both groups are shown in Table 1. They were very similar, except that significantly more cases than controls had old sensory impairment (SI) at diagnosis (duration >6 months); for old motor impairment (MI) the difference was not significant.

**Table 1:** Characteristics and outcome details of the subjects in the incidence cohort of the INFIR Cohort Study (*N* = 188).

Variable	Cases# (N = 74)		Controls (N = 73)		*p* - value*
	Frequency	Percentage	Frequency	Percentage	
**Sex**					
Men	52	70.3	51	69.9	0.96
Women	22	29.7	22	30.1	
**Age group**					
12–20	7	9.4	13	17.8	0.035
21–30	21	28.4	27	37.0	
31–40	17	23.0	20	27.4	
41–50	25	33.8	9	12.3	
>50	4	5.4	4	5.5	
**Classification**					
BT	46	62.1	43	58.9	0.12
BT (PN)**	3	4.1	0	0	
BL (PN)***	0	0	4	5.5	
BL	18	24.3	17	23.3	
LL	7	9.5	9	12.3	
**Smear BI** ****					
Positive	23	31.1	24	32.9	0.82
Negative	51	68.9	49	67.1	
**Old SI** ˆ					
Yes	37	50.0	20	27.4	0.0049
No	37	50.0	53	72.6	
**Old MI** ˆˆ					
Yes	9	12.2	4	5.5	0.15
No	65	87.8	69	94.5	
	*N* = 188 ˆˆˆ				
**Type 1 reaction**					
Skin only	12	6.4			
Skin+neuritis	3	1.6			
Skin+NFI	2	1.1			
Skin+NFI+neuritis	2	1.1			
All T1R	19	10.1			
**Type 2 reaction**					
Skin only	3	1.6			
Skin+neuritis	1	0.5			
Skin+NFI	1	0.5			
Skin+NFI+neuritis	0				
All T2R	5	2.7			
**NFI only**					
MI+SI	4	2.1			
MI only	4	2.1			
SI only	36	19.1			
All NFI	44	23.4			
**Neuritis only**					
no NFI	1	0.5			
With NFI	5	2.7			
All neuritis	6	3.2			
**Any event**	74	39.4			

# cases = patients with sensory impairment by monofilament test, motor impairment by VMT or a Type 1 or 2 leprosy reaction; ^*^ = Chi-square test; ^**^ = Pure neuritic, BT histology; ^***^ = Pure neuritic, BL histology; ^****^ = Bacteriological index of the skin smear at diagnosis; ˆ = Sensory impairment; ˆˆ = Motor impairment; ˆˆˆ = the cumulative incidence of reactions and nerve function impairment (NFI) used the whole cohort as denominator (*N* = 188).

Nearly 75% of patients with an outcome event had their first event within the first six months. Eleven had an event during the second half of the first year (15%); the remaining 8 occurred during the second or even third year. NFI without skin signs of reaction was the most frequent outcome event (23% of the cohort; 60% of all events). T1R came second with 10% (Table 1). SI was much more frequent than motor impairment (MI; 19% vs. 2.1%). Isolated MI also occurred in 2.1%.

### Comparing nerve function tests

Table 2 shows the relative frequencies of impairment as detected by the various tests among patients who had an outcome event (N = 74), compared to the results among the matched controls. Nerve conduction parameters, particularly SNAP amplitudes and CMAP velocities, were the most frequently affected measurements, followed by thermal thresholds, VPTs, MFs and, lastly, VMTs. Regarding ulnar nerves, SNAP amplitude was impaired in 42%, CMAP velocity in 43%, WDT in 29%, CDT in 13%, VPT in 19%, MFT in 15% and VMT in 11%. In the radial cutaneous nerve, SNAP amplitude was impaired in 60%, WDT in 48%, CDT in 42%, VPT in 24% and MFT in 8.8%. The pattern was not always completely consistent. In the median nerve, WDTs were more often impaired than NC parameters, while in the sural nerve, NC was as often impaired as thermal sensation.

**Table 2:** Relative frequencies of neural impairment, compared to age and sex-specific normal thresholds, according to the various nerve function tests used in the INFIR Cohort Study at the time of the incident event.

Test*	Ulnar				Median				RC				Lat pop				PT				Sural			
	Cases		Controls		Cases		Controls		Cases		Controls		Cases		Controls		Cases		Controls		Cases		Controls	
	n	%	n	%	n	%	n	%	n	%	n	%	n	%	n	%	n	%	n	%	N	%	n	%
**MFT** (N)	148		146		148		146		148		146		148		146		146		146		148		146	
All impairment	22	14.9	6	4.1	13	8.8	2	1.4	13	8.8	3	2.0					68	46.6	27	18.5	55	37.2	26	17.8
New impairment	10	6.8	0		7	4.7	0		4	2.7	0						48	32.4	0		9	6.1	0	
**VMT** All	16	10.8	6	4.1	2	1.4	0						4	2.7	1	0.7								
New	6	4.1	0		1	0.68	0						4	2.7	0									
**SNC** (N)	138		129		135		130		129		122										134		125	
No conduction	34	24.6	15	11.6	18	13.3	7	5.4	37	28.7	22	18.0									74	55.2	49	39.2
Latency (msec)	48	34.8	18	14.0	36	26.7	13	10.0	51	39.5	32	26.2									60	44.8	41	33.3
Amplitude (µV)	58	42.0	32	24.8	40	29.6	28	21.5	77	59.7	60	49.2									92	68.7	79	63.2
**MNC**	Wrist												Ankle											
(N)	132		129		129		138						133		131									
No conduction	3	2.3	3	2.3	1	0.8	1	0.7					16	12.0	10	7.6								
Latency (msec)	17	12.9	13	10.1	8	6.2	5	3.6					30	22.6	19	14.5								
Amplitude (mV)	22	16.7	21	16.3	19	14.7	16	11.6					42	31.6	35	26.7								
	Elbow												Fibula											
(N)	127		128		126		126						134		131									
No conduction	3	2.4	1	0.8	1	0.8	1	0.8					17	12.7	7	5.3								
Amplitude (mV)	35	27.6	28	21.9	20	15.9	20	15.9					43	32.1	33	25.2								
Velocity (m/sec)	54	42.5	40	33.3	32	25.4	20	15.9					39	29.1	28	21.4								
**WDT** (N)	144		138		143		138		143		136						143		138		143		138	
	42	29.2	32	23.2	59	41.3	39	28.3	68	47.6	69	50.7					89	62.2	53	38.4	96	67.1	68	49.3
**CDT**	19	13.2	14	10.1	27	18.9	21	15.3	59	42.3	40	29.2					57	40.1	31	22.5	83	58.5	49	35.4
**VPT** (N)	146		144		145		144		144		142						143		142		144		144	
	28	19.2	11	7.6	27	18.6	13	9.0	34	23.6	23	16.2					46	32.2	28	19.7	49	34.0	30	20.8

Cases are patients with an incident event (sensory impairment by monofilaments, motor impairment by VMT or a Type 1 or 2 leprosy reaction; N = 74); controls are patients with leprosy, but without an event (N = 73). Frequencies among the control are prevalences of impairment at the same follow-up visit as the incident event of their respective controls. MFT = monofilament test, VMT = voluntary muscle test, MNC = sensory nerve conduction, WDT = warm detection threshold, CDT = cold detection threshold, VPT = vibration perception threshold.

Electrophysiology and QST detected impairment more often than MFT and VMT (Table 2). In the ulnar nerve, MFs detected 15% impairment, while SNC detected 42%; in the sural nerve, 37% and 69%. VMT detected even less impairment than MFT (11% vs. 43% for the ulnar and 2.7% vs. 32% for lateral popliteal). It was striking that there were relatively small differences in impairment frequency between cases and controls. For some nerves, these differences were not even statistically significant (e.g. ulnar MNC velocity 42.5% vs. 33.3%).

Table 3 shows the relationship between duration of onset of clinically detectable NFI (MFT and VMT) and the duration of impairment as measured with neurophysiological tests. The majority of nerves that were impaired by the monofilament test (both old or new impairment), already had evidence of old impairment by SNC or WDT (onset >6 months) (e.g. ulnar and sural nerves 100%). In addition, up to 12% of ulnar and 8% of sural nerves with normal monofilament tests had new impairment of SNC latency or amplitude. This was even true for nerves in the control group. Hardly any of the new impairment detected by SNC was detected by MFT (ulnar 7.7%; median 9%, RC 0%), except in the sural (43%). For WDT these figures were only slightly better (ulnar 29%, median 10%, RC 0%, PT 46% and sural 23%). MFT and CDT and VPT gave better concordance for new impairment detection, although there was more new MFT impairment detected that was not picked up by CDT or VPT. Most of the new CDT and VPT impairment in the arms was not picked up by MFT (as old or new).

**Table 3:** Cross tabulation of sensory impairment, compared to age and sex-specific normal thresholds, between the monofilament test and the various other nerve function tests used in the INFIR Cohort Study at the time of the incident event in the respective nerves.

Test*	Ulnar					Median					Radial Cutaneous					Posterior Tibial					Sural				
	Cases			Controls		Cases			Controls		Cases			Controls		Cases			Controls		Cases			Controls	
	No	Old	New	No	Old	No	Old	New	No	Old	No	Old	New	No	Old	No	Old	New	No	Old	No	Old	New	No	Old
**MFT** Imp. status	126	12	10	140	6	135	6	7	144	2	135	9	4	143	3	78	20	48	119	27	93	46	9	120	26
%	85.1	8.1	6.8	95.9	4.1	91.2	4.1	4.7	98.6	1.4	91.2	6.1	7.0	98	2.0	53.4	13.7	32.9	81.5	18.5	62.8	31.1	6.1	82.2	17.8
**SNC** (N) ˆ	113	11	9	122	6	122	6	6	127	2	114	8	3	117	3						81	42	9	99	24
No conduction	10	10	9	9	5	10	4	3	5	1	24	8	1	20							24	38	8	24	23
%	8.8	91	100	7.3	83	8.2	67	50	3.9	50	21	100	33	17							30	95	89	24	96
Latency old*	7	11	8	5	5	10	5	2	2	1	21	8	1	19							13	30	4	14	19
%	6.5	92	89	4.1	83	8.6	83	40	1.6	50	19	100	25	17							17	71	50	14	83
Latency new*	12		1	6		10		1	6		11			9							4	2	1	6	
%	11		11	5.0		8.6		20	4.8		10			7.8							5.1	4.8	13	6.1	
Amplitude old	21	11	9	12	5	18	5	3	16	2	50	8	3	48	3						31	40	9	37	23
%	19	100	100	10	83	16	83	60	13	100	47	100	100	42	100						39	98	100	39	100
Amplitude new	7			10	1	5			4		4			5							6	1		11	
%	6.5			8.4	17	4.4			3.3		3.8			4.4							7.6	2.4		12	
**WDT** (N)	116	10	7	126	2	117	4	5	124	1	111	3	3	109	1	63	14	35	108	10	71	36	9	103	10
Old*	10	5	2	6	1	8	3	1	5	1	24	2	2	19	1	12	10	23	21	6	18	31	7	28	9
%	8.6	50	29	4.8	50	6.8	75	20	4.0	100	22	67	67	17	100	19	71	66	19	67	25	86	78	27	90
New*	10	1	3	15		27	1	2	20		14			23		7	2	4	4	2	10	2	1	5	1
%	8.6	10	43	12		23	25	40	16		13			21		11	14	11	3.7	22	14	5.6	11	4.9	11
**CDT** (N)	119	10	8	130	1	123	5	6	126	2	116	5	3	118	2	68	16	34	112	11	76	33	9	108	9
Old*		6	2	1	1	2	3	2	2		16	3	2	12	1	7	7	10	9	5	11	30	5	15	7
%		60	25	0.8	100	1.6	60	33	1.6		14	60	67	10	50	10	44	29	8.0	45	14	91	56	14	88
New*	2		2	5		10		1	9	1	18		1	10		1	2	6	2		8	2	3	6	
%	1.7		25	3.9		8.1		17	7.1	50	16		33	8.5		1.5	13	18	1.8		11	6.1	33	5.6	
**VPT** (N)	122	10	6	136	3	124	6	7	137	2	122	5	4	128	1	71	16	41	114	13	81	38	9	115	14
Old*	6	5	2	5		6	4	1	8		8	3		6		5	6	9		10	4	12	1	2	7
%	4.9	50	33	3.7		4.8	67	14	5.8		6.6	60		4.7		7	38	22		82	4.9	32	11	1.7	54
New*	5		2	1		6		2			8	1	1	4		6	2	4	2	1	10	4	2	6	
%	4.1		33	0.7		4.8		29			6.6	20	25	3.1		8.5	13	9.8	1.8	9.1	12	11	22	5.2	

Cases are patients with an incident event (sensory impairment by monofilaments, motor impairment by VMT or a Type 1 or 2 leprosy reaction;an incident event (N = 74); controls are patients with leprosy, but without an event (N = 73). Frequencies of impairment among the controls refer to the same follow-up visit as the incident event of ‘their cases’. MFT = monofilament test, WDT = warm detection threshold, CDT = cold detection threshold, VPT = vibration perception threshold; ˆ the N represents the denominator for the first parameter listed. However, due to a variety of reasons, the N was not always exactly the same for each parameter of that same test; ^*^ Old = impairment already present at diagnosis; New = new or additional impairment at the time the patient was diagnosed to have an outcome event; ^**^ w/a = at wrist or ankle; e/f = at elbow or fibula head.

Of the old NC and thermal sensory impairment, some was also detected as old by MFT (e.g. ulnar amplitude 27%; sural amplitude 50%); however, a substantial proportion was detected as new MFT impairment (ulnar 22%; sural 11%) or was not detected at all (ulnar 51%; sural 39%). NC and thermal testing (particularly WDT in the arms) picked up most nerves with new impairment among the control group (up to 21% for WDT in the radial cutaneous nerve).

A similar pattern was seen when comparing impairment detected by MNC and VMT (Table 4), although numbers were much smaller. There were few instances of new loss detected by VMT, the majority of which showed abnormal MNC parameters more than 6 months earlier or at the time of diagnosis. None of the new motor impairment detected by MNC was detected by VMT. Only some old MNC impairment was detected as new (ulnar 12%; lateral popliteal 10%).

**Table 4:** Cross tabulation of motor impairment, compared to age and sex-specific normal thresholds, between the VMT and various motor nerve conduction tests used in the INFIR Cohort Study at the time of the incident event in the respective nerves.

Test*	Uln					Med					LP				
	Cases			Controls		Cases			Controls		Cases			Controls	
	No	Old	New	No	Old	No	Old	New	No	Old	No	Old	New	No	Old
**VMT** Imp. status	132	10	6	141	5	144	1	1	146		144		4	145	1
%	89.1	6.8	4.1	96.6	3.4	98.6	0.7	0.7	100		97.3		2.7	99.3	0.7
**MNC** w/a** (N) ˆ	122	6	4	124	5	128	1		138		128		3	127	1
No conduction	1	2		2	1	1			1		12		2	6	1
%	0.8	33		1.6	20	0.8			0.7		9.4		67	4.7	100
Latency old*	1	3		0	2	2					11		1	7	1
%	0.9	60			67	1.6					9.2		33	5.7	100
Latency new*	4			6		2			4		6		1	5	
%	3.5			5.0		1.6			2.9		5.0		33	4.0	
Amplitude old*	1	4		3	3	5			2		24		3	18	1
%	0.9	67		2.5	100	4.1			1.5		19		100	15	100
Amplitude new*	7	1		10		7			10		10			6	
%	6.1	17		8.3		5.8			7.5		8.0			5.0	
															
e/f** (N)	117	6	4	123	5	125	1		125		128		4	130	1
No conduction	1	2			1	1					12		3	6	1
%	0.9	33			20	0.8					9.4		75	4.6	100
Velocity old*	21	4	2	16		6			1		12		2	11	1
%	20	67	67	15		5.0			0.8		9.9		67	9.0	100
Velocity new*	16			8	1	19	1		14		15			8	
%	15			7.3	33	16	100		12		12			6.6	
Amplitude old*	10	5	2	7	3	5			3		26		3	24	1
%	9.4	83	67	5.8	100	4.2			2.5		21		75	19	100
Amplitude new*	7			13		8			9		10			2	
%	6.5			11		6.8			7.6		7.9			1.6	

Cases are patients with an incident event (sensory impairment by monofilaments, motor impairment by VMT or a Type 1 or 2 leprosy reaction; N = 74); controls are patients with leprosy, but without an event (N = 73). Frequencies among the controls refer to the same follow-up visit as the incident event of ‘their cases’. VMT = voluntary muscle test, MNC = sensory nerve conduction; ˆ the N represents the denominator for the first parameter listed. However, due to a variety of reasons, the N was not always exactly the same for each parameter of that same test; ^*^ Old = impairment already present at diagnosis; New = new or additional impairment at the time the patient was diagnosed to have an outcome event; ^**^ w/a = at the wrist or ankle; e/f = at the elbow or fibula head.

The ability of the various tests to predict a MFT outcome is shown in Table 5. Because of the small number of outcome events in different types of nerves, we have pooled all sensory nerves, except for the posterior nerve, which had a sufficient number of events on its own. Specificity and negative predictive values (NPV) were high. Positive predictive values (PPV) were low for all tests. This is because almost all nerves that developed monofilament impairment already had old impairment by other parameters. PPVs were highest for the nerves in the legs, reaching close to 40% for WDT at 8 weeks prior to the MFT event. Closer to the event, PPVs were higher for cold detection, reflecting changes in CDT in the weeks prior to changes detected by MFT.

**Table 5:** Predictive value of new impairment detected by quantitative sensory testing and sensory nerve conduction testing in predicting new impairment by monofilament test at 12, 8 and 4 weeks before the event, in five sensory nerves in the INFIR Cohort Study.

			3^rd^ visit				2nd visit				1st visit				Event			
Nerve	Test*	N**	Sens	Spec	PPV	NPV	Sens	Spec	PPV	NPV	Sens	Spec	PPV	NPV	Sens	Spec	PPV	NPV
All	SNCL	18/611	11	93	4.6	97	17	93	6.8	97	16	90	5.0	97	17	91	5.3	97
sens ˆ																		
	SNCA	18/615	0	93	0	97	0	90	0	97	0	90	0	97	0	92	0	97
	WDT	19/669	16	89	3.9	97	33	84	5.5	98	26	78	3.3	97	26	80	3.6	97
	CDT	19/669	16	92	5.5	97	22	91	6.7	98	26	87	5.6	98	37	90	9.3	98
	VPT	20/699	15	93	6.3	97	11	93	4.0	97	11	93	4.0	97	30	95	14	98
PT***	WDT	37/165	19	87	29	80	29	86	39	80	26	85	34	80	24	89	39	80
PT	CDT	36/165	5.4	96	25	79	5.3	94	22	76	16	93	40	79	28	97	71	83
PT	VPT	37/174	2.6	93	10	78	11	93	31	78	16	91	32	80	14	95	42	80

Only cases (patients with sensory impairment by monofilaments, motor impairment by VMT or a Type 1 or 2 leprosy reaction) with at least a 12-week follow-up before occurrence of the event were included. ^*^ VPT = vibration perception threshold; WDT = warm detection threshold; CDT = cold detection threshold; SNC = sensory nerve conduction, L = distal latency, A = amplitude; ^**^ N = number of nerves with an sensory outcome event (monofilament) out of all nerves tested (these numbers varied at the different time points, because of occasional equipment failure, or because occasionally certain parameters could not be measured; the numbers shown refer to the number at the time of the outcome event); ˆ all sensory nerves pooled, except for the posterior tibial nerve; ^***^ PT = posterior tibial nerve.

### Comparing nerves

Sensory impairment frequencies varied considerably between nerves (Table 2). MFT impairment frequencies in these nerves were 14.9% in the ulnar, 8.8% in the median and RC, 47% in the PT and 37% in the sural nerve. SNC impairment also varied and was most frequent in the radial cutaneous and sural nerves (e.g., amplitude: ulnar 42%, RC 60% and sural 69%). For WDT impairment, these figures were 29%, 48% and 67%, respectively. CMAP velocity was impaired in 43% of ulnar, 25% of median and 29% of lateral popliteal nerves.

### Trends over time

**Figure 1:**
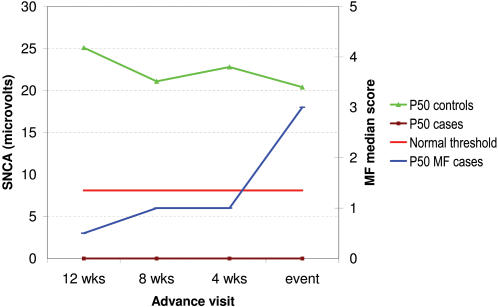
Trend in sensory amplitude in the sural nerve prior to a sensory impairment event detected by monofilaments (case nerves: n = 6; control nerves: n = 14). ‘Cases’ are nerves with new sensory impairment by monofilament (MF) test at time ‘0’; ‘controls’ are nerves without any clinically detectable sensory impairment during follow-up; P50 = median value; normal threshold refers to the specific parameter tested (here SNC amplitude).

**Figure 2:**
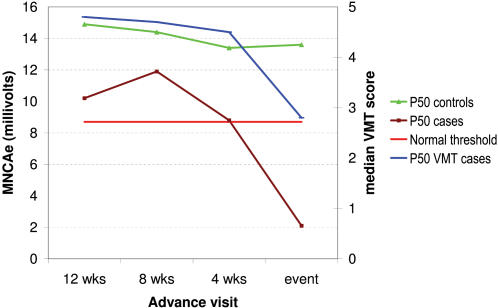
Trend in CMAP amplitude in the ulnar nerve above the elbow prior to a motor impairment event detected by VMT (case nerves: n = 5; control nerves: n = 14). ‘Cases’ are nerves with new motor impairment by voluntary muscle test (VMT) at time ‘0’; ‘controls’ are nerves without any clinically detectable sensory or motor impairment during follow-up; P50 = median value; normal threshold refers to the specific parameter tested (here MNC amplitude).

Figure 1 shows SNC results over time in sural nerves of subjects for whom at least 3 advance visits were available *and* in whom new sural MFT impairment was diagnosed (at time ‘zero’). The graphs show the median amplitude values of cases with MFT impairment and controls, as well as the normal threshold. While median values of control nerves were in the normal range, no sensory amplitudes could be recorded from any of the ‘case nerves’ even 3 visits before deterioration in function was detected by MFT test. The pattern was similar in other nerves, but there were too few impaired nerves sufficient advance visits for meaningful analysis. CMAP amplitude values of ulnar case nerves decreased sharply from 8 weeks prior to the event (Figure 2).

**Figure 3:**
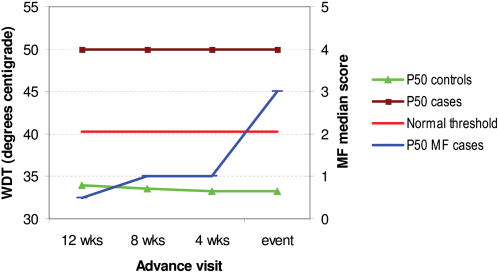
Trend in WDTs in the sural nerve prior to a sensory impairment event detected by monofilaments (case nerves: n = 8; control nerves: n = 16). ‘Cases’ are nerves with new sensory impairment by monofilament (MF) test at time ‘0’; ‘controls’ are nerves without any clinically detectable sensory impairment during follow-up; P50 = median value; normal threshold refers to the specific parameter tested (here warm detection thresholds).

WDT impairment also often started 12 weeks or more before the MFT impairment became detectable (Figure 3). Control nerve thresholds were just below or around the normal cut-off. Impairment in cold and vibration perception generally occurred later (closer to the time of the MFT event) than was the case with SNC and WDT impairment (data not shown).

CDTs decreased slightly at the time of a MFT event, but the effect was not very pronounced, except in the radial cutaneous and sural nerves (not shown). In the latter two, the median threshold dropped 4.6 and 5.8°C four weeks before the event and another 9 and 8.1°C at the time of the event. No downward trend was observed in the control nerves.

VPTs were generally within the normal range, right up to a MFT impairment event (not shown). An increase in median threshold occurred in all nerves at the event, but this was statistically significant only for the ulnar nerve (*p* = 0.047, Wilcoxon test). The trend pattern in the three dynamometry measures was variable, but no downward trend was observed until 4 weeks prior to a VMT event in the hand (not shown). At the time of the outcome event, a clear decrease was observed in grip strength, similar to the one seen in VMT sum score. No trend was obvious in either key-pinch or pulp-to-pulp pinch strengths (not shown).

## Discussion

This study investigated which test or combination of tests would be the earliest in detecting changes in nerve function prior to a clinical nerve damage event. This is the first study to examine this question prospectively, using instruments that assess thick myelinated, thin myelinated, as well as unmyelinated fibre systems.

The results have been very revealing. Electrophysiology and QST detected far more sensory and motor neuropathy in this cohort of leprosy patients than the standard tests, MFT and VMT. Sensory nerve conduction (particularly amplitude) and warm perception testing proved by far the most sensitive in picking up sub-clinical neuropathy ahead of a deficit becoming detectable by MFT. In the ulnar nerve, SNAP amplitudes and WDTs were abnormal in 42% and 30% of cases, while monofilaments showed impairment only in 15%. For the sural nerve, these figures were 69%, 67% and 37%, respectively. Even 12 weeks before the onset of a MFT sensory event, SNC and WDTs were already abnormal in nerves that would develop a MFT impairment (see Figure 1 and Figure 3). Because most outcome events occurred in the first few months following the start of MDT, it proved impossible to trace back to the onset of warm detection impairment. The same was true for SNC; both distal latency and amplitude were abnormal at least 12 weeks before sensory impairment became clinically evident. This corresponds with findings in earlier cross-sectional NC studies that a substantial proportion of clinically unaffected nerves have evidence of sub-clinical neuropathy [19],[23],[24],[26]. Tzourio et al. [45] and Antia et al. [20] found NC abnormalities in patients with very early forms of leprosy, but did not relate this to the occurrence of subsequent NFI. Previous workers have found that SNAP amplitudes were more severely affected than latencies or velocities [24], and may indicate early nerve involvement. Of the two thermal modalities, warm perception was affected much earlier than cold perception, although the predictive value of both tests was very similar. This in contrast to diabetic neuropathy, which affects cold sensation more than warm sensation [31].

In most of the nerves in which a new MFT events occurred during follow-up, SNC or WDT impairment of long duration was already present (ulnar 100%, PT 68%, sural 78%; see Table 3). Very little of the new impairment detected by SNC or WDT was clinically detected with MFT. In a small proportion of posterior tibial nerves (23%), new clinical impairment occurred without evidence of impairment of thermal sensation or vibration sense. This has been described previously [46],[47].

The early changes were less obvious with MNC (Figure 2), although MNC parameters were frequently impaired (ulnar 32%, LP 32%). CMAP velocities and amplitudes were lower in most case nerves than in control nerves at least from 12 weeks before the event, but they were above or near the normal threshold in about half of the nerves. While vibrometry trends tended towards impairment at the time of an event, all nerves in our control sample stayed within the normal range. With dynamometry, only grip strength deteriorated towards the time of a VMT outcome event.

These data show that sensory and motor impairment detected by MFT and VMT are only the tip of an ‘iceberg of neuropathy’ in leprosy. Even in patients with no clinical signs of reaction, no nerve tenderness, no clinically evident NFI (detectable by MFT or VMT), or symptoms of nerve pain, definite evidence of silent sensory and/or motor neuropathy was found (ulnar up to 26%, RC 50%, and sural 47%). Early detection of such neuropathy is possible with a combination of SNC and WDT testing, but the prognostic benefits of this still need to be determined through a controlled treatment trial. SNC is technically difficult to test in many leprosy endemic countries, but simpler techniques, using cheaper equipment, are being developed (Wilder-Smith, personal communication). WDT measurement was technically very easy with the user-friendly TSA II equipment, but the machine is expensive and required regular maintenance and replacement of parts. A simpler, low(er)-cost machine is also being developed. A major practical problem with these tests is that some environmental temperature control is required. With SNC, changes in outside temperature can be accounted for by adjustment of the results. The TSA II required operating temperatures of less than 26°C, so an air-conditioned environment would be essential in most leprosy endemic countries.

In this study, vibrometry and dynamometry did not detect sensory and motor neuropathy before MFT and VMT. The former has been shown to be useful in monitoring diabetic neuropathy and in early detection of toxic neuropathies, such as cisplatin-induced sensory neuropathy [48]–[50]. Perhaps the testing technique – applying the vibration stimulus to soft tissue rather than superficial bony structures – accounts for the difference. The technique was chosen because, in leprosy, deep sensation is often intact when skin sensation is already impaired. Another possible reason is the relative large diameter of the vibrometer probe, which stimulates an area of several square centimetres. Because leprosy neuropathy is not homogeneous, remaining intact sensory receptors may detect the stimulus, giving the impression of normal vibration sense.

A high prevalence of impairment was found in the radial cutaneous and sural nerves (up to 60% and 69%, respectively, depending on the parameter tested). For sensory amplitude and warm sensation, the sural was the most frequently affected of all nerves. In current clinical practice, these two nerves are often not examined. Our results, as well as those of the baseline analysis of the INFIR Cohort Study [51], indicate that examination of these nerves could be important in diagnosing leprosy and monitoring nerve damage. Examining both nerves using monofilaments is easy and quick to do.

Although sensory and motor neuropathy was much more widespread than the NFI detected by MFT and VMT, these tests were still validated by the current results. MFT results correlated well with the overall level of neural impairment and changes in MFT scores mirrored changes in one or more neurophysiological parameters, particularly cold perception and VPTs. Monofilaments have been widely promoted as accurate and reliable instruments for monitoring sensory neuropathy, particularly in leprosy and diabetes [25], [52]–[58]. Samant et al. found that a combination of MFT and nerve palpation detected nearly as much ‘nerve involvement’ (33%) as did SNC testing on its own (41%), but it was not clear from the report whether this concerned the same nerves nor whether this concerned old or new impairment [26]. Breger compared MFT and SNC and found 81% concordance between MFT results and SNAP amplitudes in a sample of 142 ulnar and median nerves [47]. In the present study this particular concordance was 74% for the ulnar and 76% for the median nerve at the time of the outcome event (data not shown). Therefore, graded monofilaments and manual voluntary muscle testing do reflect overall nerve function at a given point in time, but, compared to nerve conduction or warm perception, monofilament testing underestimated the extent of the damage and detected it late. Research into neuropathy in leprosy or the treatment of such neuropathy should include SNC and/or WDT testing to detect and monitor sensory impairment not detectable by monofilaments. Further studies should investigate the prognostic value of early diagnosis and treatment of sensory impairment detected with nerve conduction and thermal testing.

## Conclusions

1. Leprosy neuropathy is much more extensive than indicated when MFT and VMT were used.
2. SNC measurements, in particular SNAP amplitude, and warm perception are the most frequently and earliest affected parameters. These are the most promising tests for early detection of leprosy neuropathy.
3. SNC parameters and WDTs often become abnormal 12 weeks or more before NFI can be diagnosed by MFT.
4. Changes in the MFT and VMT scores mirror physiological changes in affected nerves, confirming their validity as screening tools.
5. The radial cutaneous and sural nerves are affected frequently. Routine inclusion of these in assessment will help in the monitoring of leprosy nerve damage.

## Supporting Information

Text S1 Appendix with outcome definitions and diagnostic cut-offs
(0.02 MB DOC)
