# Supplementary material for: Early Diagnosis of Neuropathy in Leprosy—Comparing Diagnostic Tests in a Large Prospective Study (the INFIR Cohort Study)
Source: PLoS Negl Trop Dis. 2008 Apr 2;2(4):e212. doi: 10.1371/journal.pntd.0000212 (PMC2270341; doi:10.1371/journal.pntd.0000212)
Supplement: Text S1 — Appendix with outcome definitions and diagnostic cut-offs (0.02 MB DOC) [file pntd.0000212.s001.doc]

**Text S1**

### Outcome definitions and diagnostic cut-offs

**Sensory impairment**

A patient was diagnosed as having sensory impairment in any of the following situations:

The monofilament threshold is increased by three or more levels (filaments) on any site, OR two levels on one site AND at least one level on another site, OR one level on three or more sites for one nerve.

**Motor impairment**

A patient was diagnosed as having motor impairment if the VMT score for any muscle is less than four on the 0-5 (modified) MRC scale.

**New additional sensory or motor impairment**

Where the baseline showed ***partial or full longstanding*** impairment for two or more consecutive assessments, then if the DIFFERENCE in ‘levels’ (between now and the baseline) is 3 or more for monofilaments or 2 or more for VMT, then the patient has additional recent impairment and should be considered as having an outcome event.
